# Verbal memory and search speed in early midlife are associated with mortality over 25 years’ follow-up, independently of health status and early life factors: a British birth cohort study

**DOI:** 10.1093/ije/dyw100

**Published:** 2016-08-06

**Authors:** Daniel Davis, Rachel Cooper, Graciela Muniz Terrera, Rebecca Hardy, Marcus Richards, Diana Kuh

**Affiliations:** MRC Unit for Lifelong Health and Ageing at UCL, 33 Bedford Place, London WC1B 5JU, UK

**Keywords:** Birth cohort, life course epidemiology, cognition, verbal memory, search speed, mortality

## Abstract

**Background:**

Cognitive capabilities in childhood and in late life are inversely associated with mortality rates. However, it is unclear if adult cognition, at a time still relatively free from comorbidity, is associated with subsequent mortality, and whether this explains the associations of early life factors with adult mortality.

**Methods:**

We used data from the MRC National Survey of Health and Development, a birth cohort study prospectively assessing 5362 participants born in 1946. The present analysis includes participants followed up from age 43 and undergoing cognitive assessment (verbal memory and search speed). Mortality outcomes were notified through linkage with a national register. Cox regression was used to estimate mortality hazards in relation to cognitive performance at age 43, adjusting for early life factors, socioeconomic position and health status.

**Results:**

Data were available on 3192 individuals. Univariable analyses indicated that adult verbal memory and search speed, parental factors, childhood cognition and educational attainment were associated with mortality. However, multivariable models showed that the mortality associations with earlier life factors were explained by adult cognitive capability. A standard deviation increase in verbal memory and search speed scores was associated with lower mortality rates [hazard ratio (HR) = 0.86, 95% confidence interval (CI) 0.77-0.97, *P* = 0.02; HR = 0.88, 95% CI 0.78-1.00, *P* = 0.05, respectively), after adjustment for adult health.

**Conclusions:**

Cognitive capability in early midlife was inversely associated with mortality rates over 25 years and accounted for the associations of family background, childhood cognitive ability and educational attainment with mortality. These findings, in a nationally representative cohort with long-term follow-up, suggest that building cognitive reserve may improve later life health and survival chances.

## Introduction

Cognitive capabilities underpin health and well-being, and inverse relationships with mortality have been consistently described.^1–4^ Observed associations between cognition and mortality may have multiple explanations, perhaps varying in importance at different stages of growth, stability and decline. ^5^ Most of the literature on cognitive capability and mortality has examined the relationships when cognition is measured in childhood and early adulthood^6–8^ or in late life;^9,10–15^ few studies have measures of cognitive capabilities in child and adult life and a long mortality follow-up.

A systematic review and meta-analysis of 16 prospective studies showed each standard deviation advantage in child or adolescent cognitive ability was associated with a hazard ratio (HR) of 0.76 [95% confidence interval (CI) 0.74-0.77] for all-cause mortality.^6^ Whether this association is due to cognitive ability acting as a mediator of early adversity, a marker of ‘physiological integrity’ or a determinant of subsequent educational attainment, adult socioeconomic position and other life chances is an ongoing and policy-relevant debate;^3,16–18^ these possibilities are not mutually exclusive. In the systematic review, adjusting for SEP in childhood did not affect the HR, though accounting for adult SEP and education attenuated the estimates of effect size. These observations are consistent with findings from the Medical Research Council (MRC) National Survey of Health and Development (NSHD) demonstrating that childhood cognition explained some, but not all, of the association between childhood SEP and adult mortality in adulthood up to age 60 years, whereas education and adult SEP strongly attenuated the association.^19^

The cognition-mortality associations seen in older populations^9,12–14^ may also be driven by lifetime socioeconomic factors and/or by a range of health conditions (clinical or subclinical) including obesity, diabetes, vascular disease and dementia.^20–22^ Here, the development of common chronic pathophysiological processes (e.g. atherosclerosis, inflammation) may have an impact on cognition as well as mortality.

Are the associations found in youth or later life also evident in early midlife? Do the factors associated with mortality identified in childhood exert their influence through adult cognition, and at an age still relatively free from comorbidity? The relative impact of child and adult cognition on mortality might have implications for interventions aimed at reducing health inequalities.^23^ Similarly, whether any associations are linear (for the whole population) or only pertain to a subset of cognitive ability, is relevant from a policy perspective. Finally, associations with mortality in connection with specific cognitive domains or tasks (e.g. episodic memory or reaction speed) or specific causes of death, may suggest different underlying biological mechanisms. For example, neuroimaging studies lend some support to the hypothesis that search speed is a fundamental mental function representing brain integrity, and as such might be more closely related to mortality than other cognitive tasks.^24,25^ Cohort studies with prospective life course data, such as the NSHD, offer an opportunity to investigate how different measures of cognitive capability in early midlife are related to subsequent mortality, and whether any associations are influenced by lifetime socioeconomic circumstances, early cognitive ability or health status.^6,19^ This study set out to address the following questions.

- Are verbal memory and search speed in early midlife (at age 43) associated with all-cause mortality over the subsequent 25 years?
- To what extent are the associations of family background and childhood cognitive ability with mortality explained by cognition in early midlife?
- To what extent are any relationships between cognitive capability in early midlife and subsequent mortality explained by adult SEP or health status?
- To what extent are the relationships between cognitive capability in early midlife and subsequent all-cause mortality also seen for cardiovascular mortality and cancer mortality?

## Methods

The NSHD is a birth cohort of 5362 men and women, a socially stratified sample from a maternity survey of all births recorded during 1 week in March 1946 in England, Scotland and Wales, and followed up over 20 times since.^26,27^ Study participants were flagged for death notification on the National Health Service Central Register in 1971 at age 25.

In 1989, when participants were 43 years old, 361 (7%) of the original sample had died, 540 (10%) had permanently refused, 607 (11%) were living abroad and 90 (2%) were permanently untraced. Of the remaining 3749, 3262 (87%) were seen by a research nurse at home;^26^ the majority of these (3247) were flagged for death notification. Previous analyses have examined the representativeness of the cohort over time.^28^ Ethical approval was obtained from the Multicentre Research Ethics Committee, and written informed consent was obtained from the study member for each component of the data collection.

### Outcome

We included deaths from any cause notified from March 1989 (43 years) until March 2014 (68 years). The primary cause of death was coded using either ICD9 or ICD10 disease classifications, and mortality from cardiovascular diseases (ICD9 codes 401-454 and ICD10 codes I10-I89) and cancers (ICD9 codes 140-239 and ICD10 codes C00-C97) were distinguished from other causes.

### Verbal memory and visual search speed at age 43

All cognitive assessments were carried out by research nurses according to a standardized protocol. Verbal memory was assessed using a 15-item word learning task, where each word was presented for 2 s. The score represents the total number of words correctly recalled over three identical trials (maximum 45). The visual search speed task required participants to cross out the letters P and W, randomly embedded within a grid of other letters, in 1 min. The score represents the total number of letters searched (maximum 600), minus the number of targets missed. Of the 3247 participants, 3192 (98%) had at least one of these measures available; this is the overall sample for analysis.

### Covariates

Covariates were selected on the basis of factors previously demonstrated to influence mortality rates in adulthood.^19,28–30^ These include childhood socioeconomic conditions, cognitive development based on childhood cognitive tests, educational attainment and adult health and lifestyle factors.

Childhood socioeconomic conditions were assessed by paternal and maternal education (primary, more than primary) and father’s occupation when participants were aged 4 (or at 11 or 15 years if missing at age 4, *n* = 29). Father’s occupational class was defined according to the Registrar General’s classification (classes I and II, professional and managerial; class III, skilled non-manual and manual; and IV and V, semi-skilled or unskilled manual occupations). Childhood cognition was measured at age 8, using tests of reading comprehension, pronunciation, vocabulary and non-verbal reasoning, designed especially for the study by the National Foundation for Educational Research in England and Wales.^31^ In keeping with previous analyses, scores in each of these domains were summed (after rescaling for equal weighting of each test) and standardized to the sample.^32^ Educational attainment by age 26 was classified as: no qualification or below ordinary secondary qualifications (vocational); ordinary secondary qualifications (‘O’ levels and their training equivalents); advanced secondary qualifications (‘A’ levels and their equivalents, and higher qualifications of degree or equivalent).

Participants’ occupational class in adulthood was defined according to the Registrar-General’s classification in the same way as for father’s occupational class. Indicators of health status were obtained by the research nurse at the home visit at age 43.^33^ These included: two measurements of systolic blood pressure using a random zero sphygmomanometer (the second measure was used in this analysis); body mass index (BMI) calculated from height and weight measured by standard protocols; self-reported doctor-diagnosed cancer, stroke and diabetes; and the World Health Organization Rose angina scale.^34^

### Statistical analyses

**Figure 1:**
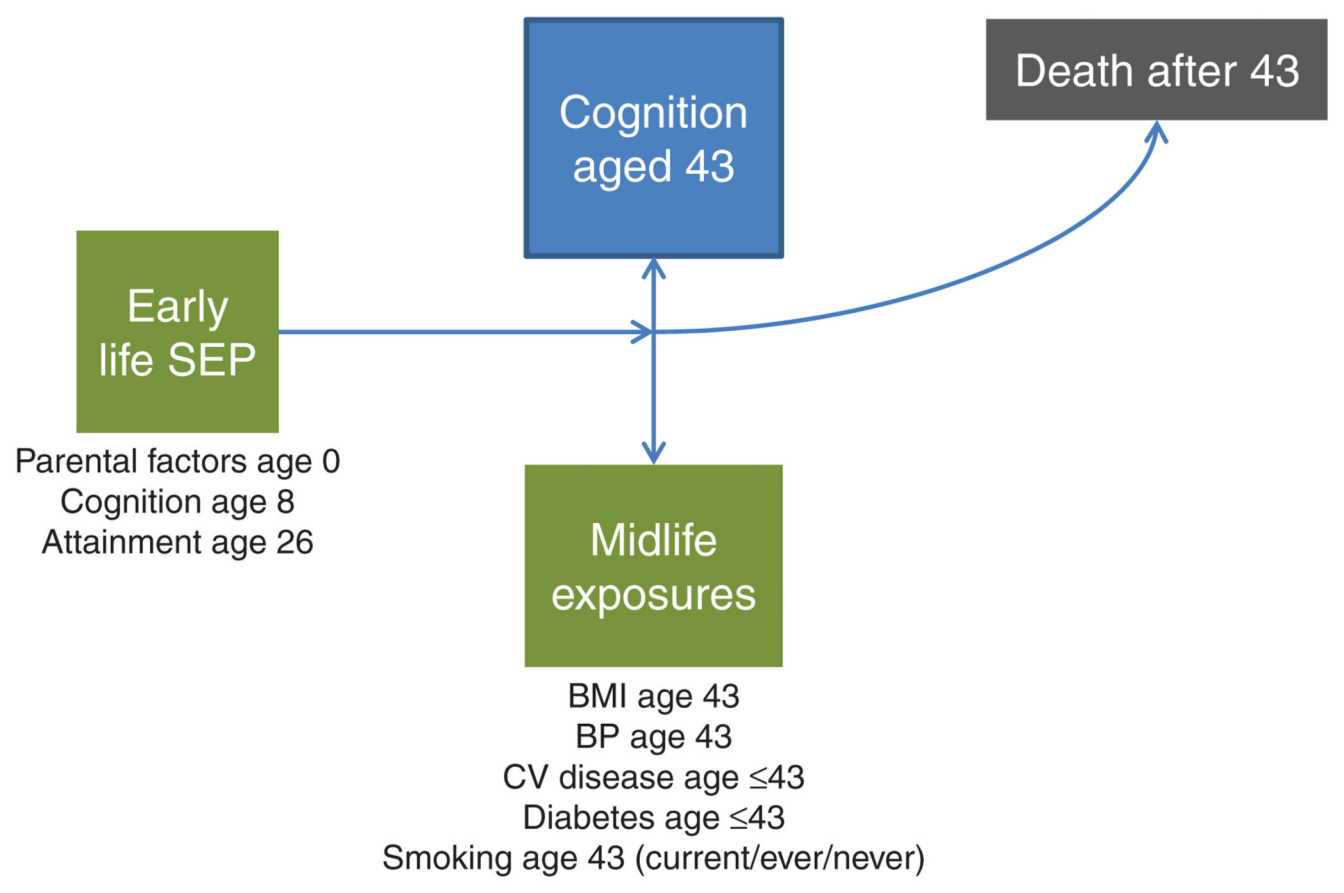
Schema outlining life course relationships contributing to analytical models.

The distribution and completeness of all variables was examined. Proportional hazards for mortality were assessed in a series of Kaplan-Meier plots and Cox regression models, where outcomes were death between ages 43 and 68 (Figure 1). Follow-up was until date of death, date of emigration, or March 2014 (if still alive and continuing to participate). Verbal memory and search speed were both standardized to have a mean of 0 and standard deviation of 1 in order to compare the effect sizes. Each analysis used cases where complete data were available for all covariates.

Correlations between the child and adult cognitive scores were examined. The relationships between both of the adult cognitive scores and subsequent mortality were first investigated in Cox regression models using the maximum available samples, and Kaplan-Meier survival curves were plotted with the scores divided by tertiles. The first series of Cox regression models tested the sex-adjusted associations between each adult cognitive score and each early life variable (parental variables, childhood cognition, education) with mortality, and then mutually adjusted in a multivariable model, using participants with complete data. Interactions between sex and cognition at age 43 were tested. A second series of multivariable models included both adult cognitive scores and the health status variables and then additionally adult occupational class. Comparison of model fit, assessed through tests of maximum likelihood, was used to identify the final model. The modelling procedures were repeated for cause-specific mortality (cardiovascular disease and cancer), using a competing risks model where deaths from other causes were censored at date of death.

Post-estimation procedures included Schoenfeld residuals for checking assumptions of proportionality. In order to assess linearity of any associations across the distribution of cognition, restricted cubic splines were fitted (five knots), plotting log-hazard ratio for mortality against z-score of cognition, and linearity visually assessed. Stata version 12.1 was used for all statistical procedures.

## Results

### Characteristics of participants

Between ages 43 and 68, 315 deaths occurred during 80 014 person-years of observation (3.9 deaths (95% CI 3.6-4.3 deaths) per 1000 person-years). Median age at death was 60 years (interquartile range 54 to 64 years). Table 1 describes the characteristics of the sample, along with the degree of missing data. At age 43, mean number of words recalled was 25/45 (SD 6.4) and mean number of letters searched was 342/600 (SD 76). The correlations between childhood cognition and verbal memory and search speed were ρ = 0.46 and ρ = 0.09, respectively. Half of the sample was male (*n* = 1599, 50%), and 1062 (33%) had educational qualifications at ‘Advanced’ level or beyond. Average BMI was 25.5 kg/m^2^ and 30% were current smokers. By age 43, few participants had been diagnosed with diabetes, stroke or cancer and less than 4% had a positive Rose angina score.

### Cognitive capability in early midlife and subsequent mortality

**Figure 2:**
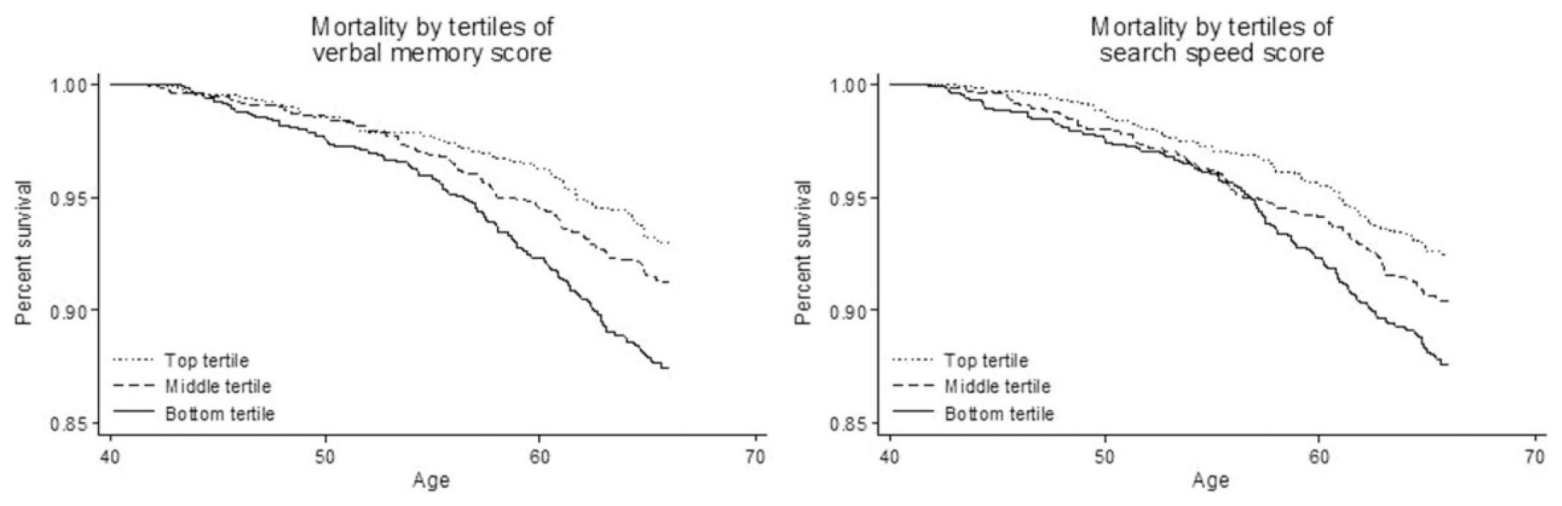
Kaplan-Meier survival curves describing unadjusted mortality from age 43 onwards in respect to tertiles of cognitive performance. Left panel: verbal memory; right panel: search speed. Note y-axis originates at 0.85.

Correlation between verbal memory and search speed at age 43 was low (ρ = 0.16). In the maximum samples (*n* = 3192), performance on verbal memory and search speed (z-scores) were both associated with mortality rate (per 1 SD increase in performance, sex-adjusted verbal memory HR 0.79, 95% CI 0.70-0.89, *P* < 0.01; sex-adjusted search speed HR 0.83, 95% CI 0.74-0.93, *P* < 0.01). Kaplan-Meier survival curves describe the association of cognitive performance with mortality from age 43, by thirds of cognitive score (Figure 2). For both z-scores in verbal memory and search speed (Figure 2), mortality rates increased with decreasing test score. This linearity in the relationship was also seen when plotting restricted cubic splines of the logHR against z-score for each cognitive performance (Supplementary figures, available as Supplementary data at *IJE* online).

### Does cognitive capability in early midlife explain the associations between early life factors and mortality?

Each adult cognitive score and each early life factor was related to mortality in the sex-adjusted models (Table 2, left column). There was no evidence of interactions between sex and the cognitive scores at age 43. In the multivariable model (Table 2, right column), the hazard ratios for the two cognitive scores were only slightly attenuated, whereas the hazard ratios for the early life variables were substantially reduced.

### Are relationships between cognitive capability in early midlife and subsequent mortality explained by adult SEP or health status?

Associations between cognitive capability at age 43 and mortality remained robust when included in a model with all other covariates independently associated with hazard for death [BMI, systolic blood pressure (BP), diabetes and smoking status] (fully adjusted verbal memory HR 0.86, 95% CI 0.77-0.97, *P* = 0.02; search speed HR 0.88, 95% CI 0.78-1.00, *P* = 0.05) (Table 3, Health Status model). In this model, other health factors (stroke, cancer, WHO angina score) were not associated with mortality. Occupational class at age 43 was not associated with mortality after adjusting for the adult cognitive scores, BMI, systolic BP, diabetes and smoking status (Table 3, SEP model); these latter variables were included in a final model (Table 3, Final model).

### Are the relationships between cognitive capability in early midlife and subsequent all-cause mortality also seen for cardiovascular and cancer mortality?

In competing risks models, verbal memory was more strongly associated with cardiovascular death rate (HR 0.79, 95% CI 0.63-0.99, *P* = 0.04, adjusted for search speed, sex, BMI, systolic blood pressure, diabetes and smoking; search speed itself was not associated with cardiovascular mortality) (Table 4). Conversely, search speed was more strongly associated with cancer mortality (HR 0.77, 95% CI 0.65-0.91, *P* < 0.01, with the same adjustments reported above, and no association with verbal memory itself and cancer mortality).

## Discussion

These analyses demonstrated an association between cognitive capability in early midlife and all-cause mortality over 25 years of follow-up, with independent effects observed for both verbal memory and search speed. Mortality associations previously shown in the NSHD with factors earlier in life, namely family background, childhood cognition and educational attainment,^16,19^ appeared to act through midlife cognitive capability at age 43. Associations remained evident after adjusting for health status and smoking, and were linear across the whole distribution of cognition, in contrast to findings on midlife physical capability and mortality in NSHD.^30^

The major strengths of these analyses are the cognitive measures from early midlife and the long, prospective follow-up of a nationally-representative population sample. They uniquely allow premature mortality from early midlife to be understood in relation to earlier measures of cognition and educational attainment. Examining a birth cohort where all participants are of the same age also allows investigation of factors unconfounded by age. Nonetheless, several limitations should be highlighted. First, in common with other cohort studies, these associations may be underpinned by residual confounding by other factors. There has been some inevitable loss to follow-up, yet the sample remains broadly representative of the census population.^35^ We adjusted for childhood cognition at age 8 as a measure of ability near the beginning of education; however, growth in cognitive capability throughout schooling and professional development may not be fully captured here. By and large, there were few missing data, but estimates may have been biased by complete case analysis. However, comparison of the estimates using a maximum sample size (sex-only adjusted model) showed little difference compared with those in Tables 2 and 3. Finally, the observed associations may be subject to secular trends and therefore specific to the generational era.^23^

Our findings are consistent with and extend other studies reporting mortality associations with cognition, by having prospective measures of cognitive capabilities in childhood and in two domains in early midlife and following up the sample for a longer duration. In the Whitehall II study, 5572 individuals were followed up from an average age in the fifth decade over 8 years, also demonstrating linear mortality associations over several domains.^36^ Our results are also comparable to analyses in the Caerphilly Prospective Study, despite the greater age of that cohort and differences in analytical approach (*n* = 1870 men age 55 to 69 at baseline for 15 years).^17^ The Lothian Birth Cohort had measures of cognition at ages 11 and 79; here, cognition in childhood appeared to have less influence than cognition assessed in older age on subsequent survival, and the change in cognition over the life course was also informative,^37^

The degree to which observed associations with mortality are specific to a cognitive domain remains unclear. The Atherosclerosis Risk in Communities (ARIC) study (*n* = 11 444, mean age 57 years, 6.3 years’ follow-up), showed that tests of speed (digit substitution) and recall (delayed word recall) were independently associated with mortality, even in this cohort with relatively little subclinical vascular disease.^38^ Participants in the Twenty-07 study (*n* = 898, mean age 56, 14 years’ follow-up) showed that general intelligence (IQ) was not associated with mortality once the estimate was adjusted for reaction time (itself robustly associated with mortality).^24^ This finding was broadly consistent with analyses in the Health and Lifestyle Survey (*n* = 6424, age range 18 to 97, followed for 21 years), but too few events occurred after measuring midlife cognition to be conclusive.^15^ However, both these contrast with a finding that verbal memory was more important when the Lothian Birth Cohort 1921 was assessed at age 79.^37^ Taken together with the analyses presented here, these differing observations may reflect underlying differences in the cohorts, the specificity of a given test for a particular cognitive domain, the age at time of testing and the degree of adjustment with other cognitive and health variables.

The underlying mechanisms that link cognition in early midlife to mortality remain largely unknown, though childhood cognition may have an impact on biological factors putatively associated with accelerated ageing.^39^ Prospective measures of SEP and health factors do not fully account for these observations, at least to the extent such variables were measured in NSHD. Both verbal memory (a largely cortical function) and search speed (with a large subcortical component) are evidently important, and we did not demonstrate primary importance for one measured domain over the other. It is unclear why these might differentially be associated with cardiovascular or cancer mortality, though we would emphasize the need for caution when interpreting these classifications. Other comparable studies have not always reported cause-specific mortality in relation to cognitive domains, and results are not necessarily consistent in magnitude or direction.^14,15,40^ Health literacy may play a part, but may itself be a marker of general cognitive capability.^41,42^ Future analysis should consider trajectories of cognitive decline from age 43 onwards, particularly if longitudinal change is even more strongly associated with widening socioeconomic inequality or change in health status.

The extent to which adult cognition is modifiable in the population has been under-explored from a policy perspective. ^43^ Nonetheless, population-based interventions to enhance cognitive capability at all stages of the life course may be beneficial at the individual and societal levels.^44,45^

In conclusion, in a large British cohort study, cognitive capability in early midlife was associated with mortality into the seventh decade of life, independently of physical health and smoking status, and accounted for the previous effects of childhood cognition, educational attainment and lifetime socioeconomic position. Overall, these findings raise interesting possibilities around building cognitive reserve into midlife, with implications for social, educational and public health policy.

## Supplementary Material

Supplementary data are available at *IJE* online.

Supplementary data

## Figures and Tables

**Table 1:** Characteristics of 3192 NSHD participants cognitively assessed at age 43 and flagged for mortality

	*N* or mean	% or SD	Missing	%
Verbal memory (raw score mean, SD)	24.7	6.4	147	4.6
Search speed (raw score mean, SD)	342	76	49	1.5
Men (*N*, %)	1599	50	0	
Father’s occupational class at child age 4			246	7.7
I + II (*N*, %)	680	21		
III NM + M (*N*, %)	1468	46		
IV + V (*N*, %)	798	25		
Father’s education by age 6, > primary (*N*, %)	1230	39	368	11.5
Mother’s education by age 6, > primary (*N*, %)	1088	34	344	10.8
General cognition age 8 (mean, SD)	0.0	1	366	11.5
Educational attainment by age 26 (*N*, %)			171	5.3
< Ordinary level (*N*, %)	1111	35		
Ordinary level (*N*, %)	848	27		
Advanced level (*N*, %)	1062	33		
**Adult factors at age 43**				
BMI (kg/m^2^) (mean, SD)	25.5	4.2	20	0.6
Systolic BP (mmHg) (mean, SD)	123	16	53	1.7
Diabetes (*N*, %)	32	1	4	0.1
Smoking status			4	0.3
Current (*N*, %)	957	30		
Ex (*N*, %)	1312	41		
Never (*N*, %)	919	29		
Positive WHO Rose angina score (*N*, %)	110	3.5	4	0.1
Stroke (*N*, %)	11	0.3	0	0.0
Cancer (*N*, %)	58	2	13	0.4
Occupational class (*N*, %)			15	0.5
I + II (*N*, %)	1382	43		
III NM + M (*N*, %)	1309	41		
IV + V (*N*, %)	486	15	15	0.5

**Table 2:** Hazard ratios for all-cause mortality by cognition at age 43 and early life factors, estimated by Cox regression. Complete case analysis of 2337 individuals

Cognition	Sex-adjusted models				Multivariable model			
		
	HR	95% CI		*P*	HR	95% CI		*P*
Verbal memory age 43 (per SD)	0.73	0.64	0.83	<0.01	0.76	0.65	0.89	<0.01
Search speed age 43 (per SD)	0.80	0.70	0.91	0.01	0.86	0.75	0.98	0.02
Sex (F cf. M)	0.76	0.59	0.98	0.03	0.86	0.66	1.13	0.3
**Early life factors**								
Father’s occupational class				<0.01				0.8
I + II	[ref]				[ref]			
III NM + M	1.34	1.06	1.76		1.19	0.82	1.74	
IV + V	1.66	1.27	2.18		1.09	0.70	1.71	
Father’s education (primary cf. > primary)	0.71	0.54	0.92	0.01	0.83	0.59	1.15	0.3
Mother’s education (primary cf. > primary)	0.74	0.61	0.91	<0.01	1.10	0.79	1.51	0.4
General cognition age 8 (per SD)	0.81	0.69	0.94	<0.01	1.02	0.86	1.21	0.8
Educational attainment age 26				<0.01				0.7
<Ordinary level	[ref]				[ref]			
Ordinary level	0.84	0.61	1.14		0.95	0.68	1.34	
Advanced level	0.62	0.45	0.84		0.93	0.63	1.37	

Verbal memory and search speed standardized to sample undertaking testing at age 43. Univariable models are estimated in the same sample as the multivariable model (*n* = 2337). All models are adjusted by sex. Sex-verbal memory and sex-search speed at age 43 interactions were tested in the sex-only adjusted models; there was no evidence of any interactions and they were not considered further. The multivariable model also includes the earlier life factors. Schoenfeld residuals test for proportional hazards for multivariable model *P* = 0.2 M, male; F, female; cf., compared with.

**Table 3:** Hazard ratios for all-cause mortality by cognition and health and smoking status at age 43, estimated by Cox regression. Complete case analysis of 2930 individuals

Cognition	Health status model				SEP model				Final model			
			
	HR	95% CI		*P*	HR	95% CI		*P*	HR	95% CI		*P*
Verbal memory age 43 (per SD)	0.86	0.76	0.97	0.02	0.89	0.79	1.02	0.09	0.86	0.76	0.97	0.01
Search speed age 43 (per SD)	0.88	0.78	1.00	0.05	0.89	0.79	1.01	0.07	0.88	0.78	1.00	0.05
Sex (F cf. M)	0.86	0.67	1.10	0.2	0.81	0.63	1.04	0.1	0.87	0.68	1.11	0.3
**Health**												
BMI (per 1 kg/m^2^)	1.03	1.00	1.05	0.06	1.03	1.00	1.05	0.07	1.03	1.00	1.06	0.05
Systolic BP (per mmHg)	1.01	1.01	1.02	<0.01	1.01	1.01	1.02	<0.01	1.02	1.01	1.02	<0.01
Diabetes (yes cf. no)	3.17	1.66	6.06	<0.01	3.30	1.73	6.27	<0.01	3.25	1.71	6.17	<0.01
Smoking status at age 43				<0.01				<0.01				
Current	[ref]				[ref]				[ref]			
Ex	0.53	0.41	0.69		0.55	0.42	0.71		0.53	0.40	0.69	<0.01
Never	0.40	0.29	0.56		0.41	0.29	0.57		0.40	0.28	0.55	<0.01
WHO Rose angina score (positive cf. negative)	1.39	0.84	2.28	0.2								
Stroke (yes cf. no)	1.72	0.42	6.95	0.5								
Cancer (yes cf. no)	1.09	0.48	2.46	0.8								
Occupational class age 43								0.06				
I + II					[ref]							
III NM + M					1.22	0.92	1.61					
IV + V					1.41	0.99	2.02					

Verbal memory and search speed standardized to sample undertaking testing at age 43. Schonfeld residuals test of proportional hazards for final model *P* = 0.2. M, male; F, female; cf., compared with.

**Table 4:** Proportional hazards for cause-specific death, estimated by Cox regression. Complete case analysis of 2952 individuals

	Verbal memory Sex-adjusted model				Search speed Sex-adjusted model				Mutually and sex-adjusted				Mutually and sex-, BMI-, systolic BP-, diabetes-, smoking-adjusted			
				
	HR	95% CI		*P*	HR	95% CI		*P*	HR	95% CI		*P*	HR	95% CI		*P*
**Cardiovascular deaths (*n* = 89)**																
Verbal memory age 43 (per SD)	0.68	0.55	0.84	<0.01					0.68	0.54	0.84	<0.01	0.79	0.63	0.99	0.04
Search speed age 43 (per SD)					0.78	0.63	0.97	0.03	0.83	0.67	1.04	0.1	0.95	0.76	1.19	0.7
**Cancer deaths (*n* = 158)**																
Verbal memory age 43 (per SD)	0.92	0.78	1.09	0.3					0.94	0.80	1.12	0.5	0.98	0.82	1.16	0.8
Search speed age 43 (per SD)					0.75	0.64	0.88	<0.01	0.76	0.64	0.91	<0.01	0.77	0.65	0.91	<0.01

Verbal memory and search speed standardized to sample undertaking testing at age 43.

## Key Messages

- Previous studies have shown that cognitive capabilities in childhood and in late life are inversely associated with mortality rates; studies with cognitive measures in child and adult life and long mortality follow-up are rare.
- Higher cognitive capability in early midlife, as measured by verbal memory and search speed, was associated with lower mortality rates over 25 years of follow-up.
- The associations were linear across the whole range of these cognitive domains and were not explained by adjustment for socioeconomic position or health status.
- Cognitive capability in early midlife accounted for the inverse associations of family background, childhood cognitive ability and educational attainment with mortality rates.
- These findings suggest that social, educational and public health policies that build cognitive reserve could have important implications for later life health and survival.
