# Supplementary figures and images for: Verbal memory and search speed in early midlife are associated with mortality over 25 years’ follow-up, independently of health status and early life factors: a British birth cohort study
Source: Int J Epidemiol. Author manuscript; Available in PMC 2019 Jul 18. (PMC6639118; doi:10.1093/ije/dyw100)

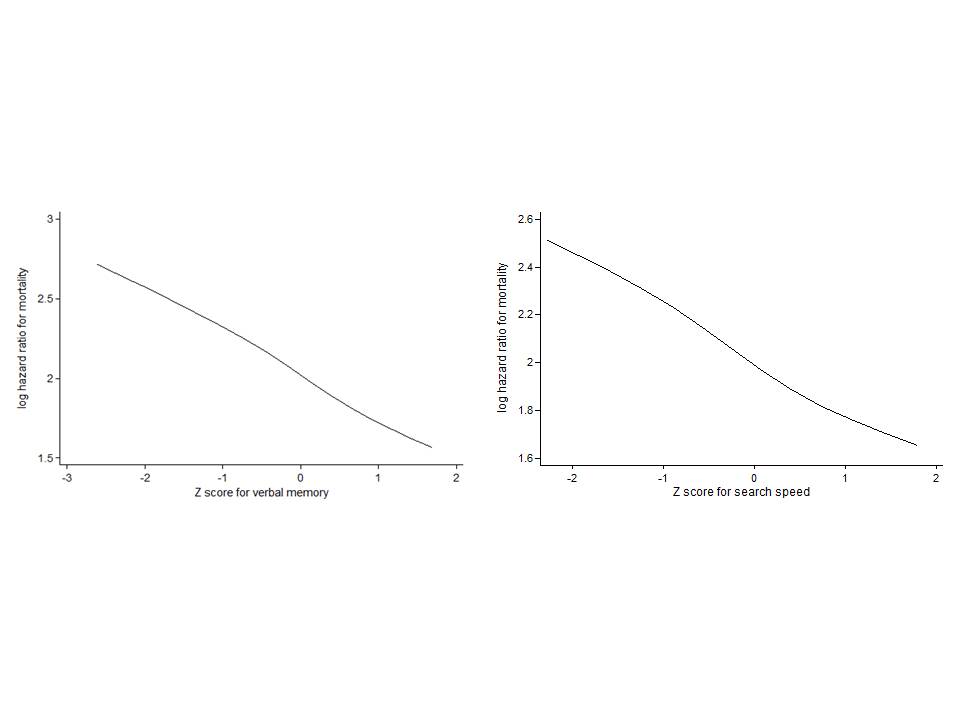

Supplement: Supplementary data [file EMS83724-supplement-Supplementary_data.zip › ije-2015-10-1426-File004.jpg]
